# Utility of Laparoscopic Approach of Orchiopexy for Palpable Cryptorchidism: A Systematic Review and Meta-Analysis

**DOI:** 10.3390/children8080677

**Published:** 2021-08-03

**Authors:** Sachit Anand, Nellai Krishnan, Zenon Pogorelić

**Affiliations:** 1 Department of Pediatric Surgery, Kokilaben Dhirubhai Ambani Hospital, Mumbai 400053, India; kanusachit@gmail.com; 2 Department of Pediatric Surgery, AIIMS, New Delhi 110029, India; nellai93@gmail.com; 3 Department of Pediatric Surgery, University Hospital of Split, 21000 Split, Croatia; 4 Department of Surgery, School of Medicine, University of Split, 21000 Split, Croatia

**Keywords:** undescended testes, palpable cryptorchidism, laparoscopy, testicular atrophy, laparoscopic orchiopexy, orchiopexy

## Abstract

Background: Traditional open orchiopexy is still a standard of treatment for palpable undescended testicles. Recently several authors reported successful results using a laparoscopic approach in the treatment of palpable cryptorchidism. The present systematic review and meta-analysis investigated the utility of laparoscopic orchiopexy for palpable cryptorchidism. Methods: Scientific databases (PubMed, Scopus, Web of Science, and EMBASE) were systematically searched for relevant articles using the following terms: (palpable cryptorchidism or palpable undescended testes) AND (laparoscopic orchiopexy or laparoscopic orchiopexy). The inclusion criteria were all children with unilateral or bilateral palpable undescended testes who underwent laparoscopic orchiopexy (LO) compared to children who underwent conventional open orchiopexy (CO). The main outcomes were the proportion of children requiring redo-orchiopexy and the incidence of postoperative complications. Secondary outcomes were duration and the cost of surgery. Results: The final meta-analysis included five studies involving 705 children; LO, *n* = 369 (52.3%) and CO, *n* = 336 (47.7%). The majority of the included patients had unilateral palpable cryptorchidism. No significant differences were found in regard to average age at the time of surgery and follow-up periods between the investigated groups. No statistically significant differences were found in regard to redo-orchiopexy rates (RR = 0.22, 95% CI 0.03–1.88, *p* = 0.17), early complications (RR = 0.66, 95% CI 0.21–2.08, *p* = 0.48) and incidence of testicular atrophy (RR = 0.36, 95% CI 0.03–3.88, *p* = 0.40). No significant differences in the operative duration were observed among the groups. Laparoscopy was associated with higher costs in most of the studies. Conclusion: LO is safe and effective in children with palpable cryptorchidism. The rates of redo-orchiopexy as well as an incidence of early complications and testicular atrophy rates are comparable to CO.

## 1. Introduction

Undescended testes (UDT) or cryptorchidism is one of the most common surgical disorders of the inguino-scrotal region in children and is one of the common causes of male infertility [1]. Recent studies have demonstrated that about 10% of infertile men have a history of UDT and orchiopexy [2]. The reported incidence of azoospermia in males with UDT is 13%; however, it can reach up to 89% in untreated cases with bilateral UDT [3]. The most recent study demonstrated that despite on-time orchiopexy, up to 25% of the children with UDT have an increased risk for infertility [4]. To identify the cases at high risk for infertility and who will require further treatment, blood sampling and testicular biopsy are required [4,5]. In addition to male infertility, as the most important consequence of cryptorchidism, boys with UDT are at a significantly higher risk of developing testicular tumors or testicular torsion, and are also more susceptible to testicular trauma compared to boys with normally positioned testicles [1,5,6,7,8]. Taking all the above-mentioned points into account, the question of the optimal time when to do orchiopexy arises. Most of the recent clinical studies indicate that orchiopexy should be performed before the first year of life [1,4,6,9,10].

Orchiopexy may be performed through a traditional open or laparoscopic approach. A traditional surgical approach for testicles palpable in the inguinal canal is open orchiopexy through the inguinal canal (Schoemaker procedure) [11]. The laparoscopic approach is frequently used only for non-palpable testes, typically located in the abdominal cavity [11,12]. About 25 years ago, the laparoscopic orchiopexy (LO) was first introduced by an American surgeon Steven Docimo for high-palpable undescended testes [13]. Subsequently, several authors have reported similar safety and efficacy with LO as compared to the conventional open orchiopexy (CO) for the management of inguinal undescended testicles [11,14,15,16,17,18,19,20,21]. They have highlighted all the benefits of minimally invasive surgery as the main advantage of LO together with the possibility of contralateral inguinal canal exploration in cases of patent processus vaginalis [11,14,15,16,17,18,19,20,21]. The rates of complications, especially in regard to testicular atrophy and ascent after surgery were similar to the open approach in the majority of the published studies [14,15,16,17,18,19,20,21].

Since so far CO has been the standard of treatment for palpable UDT and there is limited evidence to support the LO as the method of choice, the present meta-analysis investigated the utility of laparoscopic orchiopexy for palpable cryptorchidism by comparing the postoperative outcomes of both the treatment groups.

## 2. Materials and Methods

### 2.1. Data Source and Search Strategy

The present study was drafted as per the Preferred Reporting Items for Systematic Reviews and Meta-Analyses (PRISMA) guidelines [22]. A preliminary literature search was independently conducted by two authors (Z.P. and S.A.) in the PubMed database in order to confirm the absence of published meta-analyses on this topic. Subsequently, two authors (N.K. and S.A.) independently screened four scientific databases, i.e., PubMed, Scopus, Web of Science and EMBASE on 27 June 2021 (Appendix A). The search terms used were (palpable cryptorchidism OR palpable undescended testes) AND (laparoscopic orchiopexy OR laparoscopic orchiopexy). Once identified, the duplicate entries were removed, and the remaining articles were screened to select the pertinent studies as per the eligibility criteria.

### 2.2. Eligibility Criteria

The inclusion criteria for this review were: Participants—All children with unilateral or bilateral palpable undescended testes; Intervention—laparoscopic orchiopexy (LO); Comparison—children undergoing conventional open orchiopexy (CO); Outcomes—the proportion of children requiring redo-orchiopexy due to recurrence of cryptorchidism and the incidence of postoperative complications were the main outcomes studied in the present review. As a comprehensive assessment, both early and late complications (testicular atrophy) were independently assessed. In addition, a comparison was made in terms of the average operative duration and the cost of surgery among the children undergoing either LO or CO. Both these variables were considered secondary outcomes. Groups A and B included children who had undergone LO and CO, respectively.

An effort was made to include all those studies where at least one of the outcomes of interest was reported. Although the initial position of the testes (canalicular or high-inguinal or peeping testes) was not an explicit exclusion criterion; however, a separate analysis was performed to exclude the studies focusing on the extreme end of the clinical spectrum of palpable cryptorchidism (i.e., peeping testes). Case series, conference proceedings, editorials and opinion articles were excluded. The studies with unavailable full texts or in which the outcomes of interest were not reported were also excluded.

### 2.3. Data Synthesis

The data from the included studies were independently synthesized by two authors (S.A. and N.K.) using MS Excel (Version 15.24) spreadsheets. Along with the data on the study outcomes, the baseline information regarding the author’s details (name and year of publication), type of the study (retrospective, prospective cohort, or randomized controlled trial), number of patients per study, the initial position of the testes and the average age at orchiopexy was also extracted. Any disagreement among the authors was resolved through discussion with the third author (Z.P.).

### 2.4. Quality Assessment

The methodological quality assessment was independently performed by two authors (S.A. and N.K.) utilizing the Downs and Black scale [23]. This validated twenty-seven-item scale has four domains, with minimum and maximum scores of 0 and 32, respectively. Based on the assigned scores, high (0–15), moderate (16–23), or low (>23) risks of bias were assigned to the included studies. The kappa statistics were used to demonstrate the inter-observer consistency for the Downs and Black scale [24]. Based on the kappa values, the power of kappa value (or the level of agreement) was defined as poor (<0.00), slight (0.00–0.20), fair (0.21–0.40), moderate (0.41–0.60), substantial (0.61–0.80) and almost perfect (0.81–1.00).

### 2.5. Statistical Analysis

The baseline data were expressed as numbers, proportions, averages (mean or median) and ranges. The risk ratio along with 95% confidence intervals (CI) was calculated for the main (dichotomous) outcomes. Subsequently, the Mantel-Haenszel method was utilized to calculate the pooled risk ratio. The *p*-value and *I*^2^ statistics demonstrated the overall significance and heterogeneity among the included studies, respectively. A random-effects model was chosen if the heterogeneity was substantial (*I*^2^ > 50%). The meta-analysis of the comparative studies was performed using RevMan 5.4 (Cochrane Collaboration, London, UK). During the course of this meta-analysis, all the recommendations from the Cochrane handbook were followed [25]. A *p*-value of <0.05 was considered statistically significant.

## 3. Results

### 3.1. Study Characteristics

**Figure 1:**
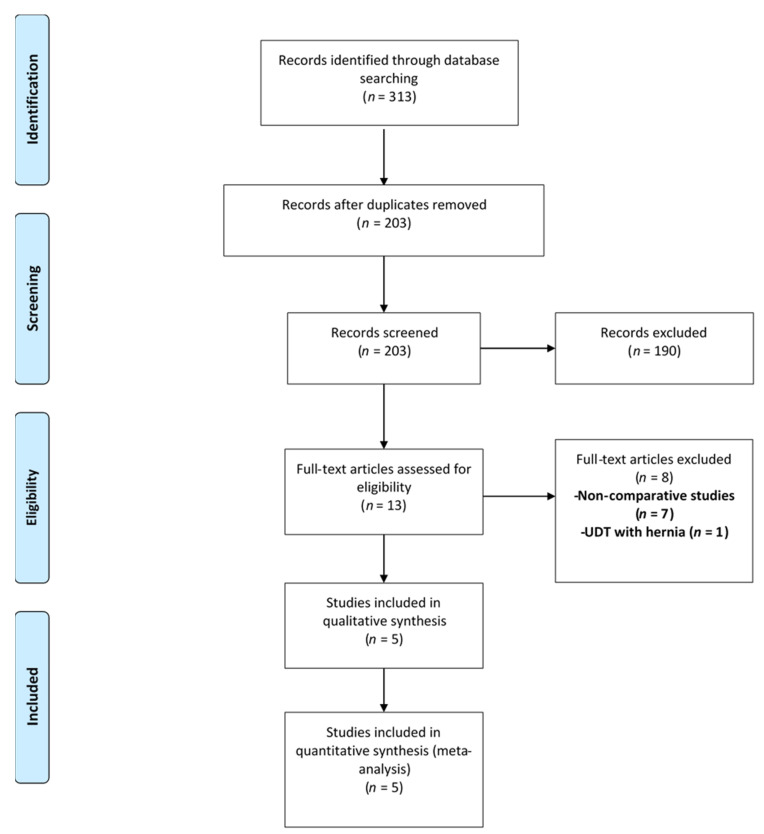
Selection of the relevant studies using the Preferred Reporting Items for Systematic Review and Meta-Analysis (PRISMA) flow diagram. Legend: UDT—Undescended testicle.

Out of 313 records identified with the search criteria, 203 abstracts were screened. Of these, 190 were excluded and 13 full-texts were assessed for eligibility. Eight studies were further excluded as they were non-comparative (*n* = 7) or dealt with palpable cryptorchidism with associated inguinal hernia (*n* = 1). Therefore, the final meta-analysis included only five studies [17,18,19,20,21]. Only one of these studies was a randomized controlled trial [18]. The rest were either prospective cohort (*n* = 2) or retrospective (*n* = 2) studies [18,19,20,21]. A total of 705 children, 369 and 336 in groups A and B, respectively, were included in these studies (Figure 1).

The baseline characteristics of the patients are depicted in Table 1. None of the studies demonstrated a difference in the average age at surgery among the two groups. The majority of the included patients had unilateral palpable cryptorchidism. Although the follow-up periods varied among different studies, there was no significant difference in the follow-up durations between the two patient groups. The comparison of average operative duration is depicted in Table 2. Apart from one study, no significant differences in the operative duration were observed among both the groups. Three studies highlighted the cost of orchiopexy via either approach. All of them demonstrated higher operative expenses associated with LO.

### 3.2. Summary of the Included Studies

#### 3.2.1. Escarcega-Fujigaki et al., 2011

In this prospective study from Mexico, the authors describe their experience of 63 children with palpable cryptorchidism. LO and CO were performed in 30 children (38 testicles) and 33 children (37 testicles), respectively. All children were discharged within 24 h. Apart from one child developing a scrotal hematoma after CO, there were no early or late postoperative complications. None of the children had recurrence of cryptorchidism. In addition, the authors utilized the visual analogue scale (VAS) to evaluate the postoperative pain. LO approach was associated with less pain as compared to CO. LO was associated with 15% additional expenses as compared to CO [19].

#### 3.2.2. Elderwy et al., 2014

In this quasi-randomized trial from Egypt, the authors included 46 children (all unilateral) with peeping testes. The median age at presentation of the cohort was 2.5 years (range 0.5–12). LO and CO were performed in 21 and 25 cases, respectively. The early postoperative complications were more prevalent in the CO group (*p* = 0.57). None of the children developed testicular atrophy. Recurrence of cryptorchidism was noticed in only two children from the CO group (*p* = 0.49). This study also utilized the visual analogue scale (VAS) for assessment of postoperative pain 6 h after the procedure. No significant difference in the intensity of pain was observed among the two treatment groups. LO was associated with 25% additional expenses as compared to CO [18].

#### 3.2.3. Saka et al., 2020

This retrospective study from Japan included 49 children (63 testicles) with palpable UDT. No differences in the age, weight and laterality of the UDT were noticed among the two treatment groups. LO and CO were performed in 24 children (33 testicles) and 25 children (30 testicles), respectively. The authors noticed a contralateral patent processus vaginalis (PPV) in 80% of the unilateral cases. All of these were closed even if they were small. There were no early or late postoperative complications in either group. None of the children developed testicular ascent (or recurrence of cryptorchidism) during the follow-up period [20].

#### 3.2.4. Yang et al., 2020

In this prospective from China, 256 children (38 bilateral) with palpable cryptorchidism were included. The median age at presentation of the cohort was 2.5 years (range 0.5–12). LO and CO were performed in 124 children (140 testicles) and 132 children (154 testicles), respectively. The early postoperative complications were more in the CO versus LO group (*p* > 0.05). Testicular ascent was observed in only two children from the CO group (*p* > 0.05); both of them required redo-orchiopexy. None of the children developed testicular atrophy. The authors concluded that the final testicular position is better after LO. LO was associated with 19% additional cost as compared to CO [21].

#### 3.2.5. Gu et al., 2021

This retrospective study was conducted in China. A total of 291 children (all unilateral) with palpable cryptorchidism were enrolled. The median age at presentation and laterality of UDT showed no significant difference among the two treatment groups. LO and CO were performed in 170 and 121 cases, respectively. None of the children developed recurrence of cryptorchidism. Testicular atrophy was noticed in three children, two from CO group and one from LO group (*p* = 1.00). The authors also studied mean operative time and normal activity time among the two treatment groups. Both were significantly shorter for the LO group as compared to the CO group. Upon subgroup analysis, the mean operative time and activity time were significantly less for children with <2 years of age. Therefore, the study concluded that LO is an appropriate approach for younger children with a patent deep ring [17].

### 3.3. Methodological Quality Assessment

The Downs and Black scores assigned to each study by both authors are demonstrated in Table 3. The mean scores ranged between 18 to 20.5. Although the study by Gu et al. had the highest score, all studies had a moderate risk of bias. Inter-rater agreement was almost perfect with a kappa of 0.926 (*p* < 0.0001).

#### 3.3.1. Meta-Analysis of Outcomes—Recurrence of Cryptorchidism and Need for Redo-Orchiopexy

**Figure 2:**
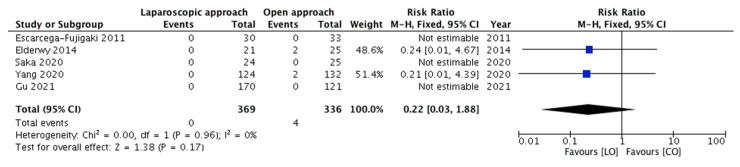
Forest plot comparison between the two treatment groups for the need for redo-orchiopexy.

All five studies, with four events, depicted the recurrence of cryptorchidism among the included children. The need for redo-orchiopexy was compared among 369 and 336 children belonging to groups A and B, respectively. The pooled risk ratio (Figure 2) for redo-orchiopexy in group A versus group B was 0.22 (95% CI 0.03–1.88), demonstrating no statistically significant difference (*p* = 0.17). For this outcome, no significant heterogeneity (*p* = 0.96; *I*^2^ = 0%) was observed among the included studies.

#### 3.3.2. Meta-Analysis of Outcomes—Incidence of Early Postoperative Complications

**Figure 3:**
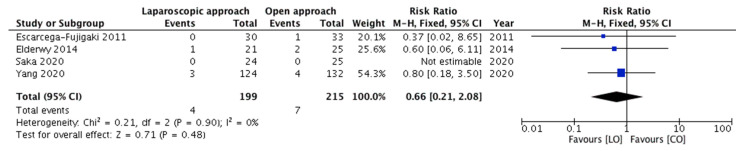
Forest plot comparison between the two treatment groups for the incidence of early postoperative complications.

Four studies, constituting 199 and 215 children from groups A and B, respectively, reported this outcome. The incidence of early complications was compared among the two groups. Complications including wound infection (port-site, inguinal or scrotal), scrotal edema and scrotal hematoma were reported in eleven patients (four and seven in groups A and B, respectively). Pooling the data (Figure 3) demonstrated no significant difference in the incidence of early complications in group A versus group B (RR = 0.66, 95% CI 0.21–2.08, *p* = 0.48). For this outcome, the estimated heterogeneity among the included studies was not statistically significant (*p* = 0.90; *I*^2^ = 0%).

#### 3.3.3. Meta-Analysis of Outcomes—Incidence of Testicular Atrophy (Late Complication)

**Figure 4:**
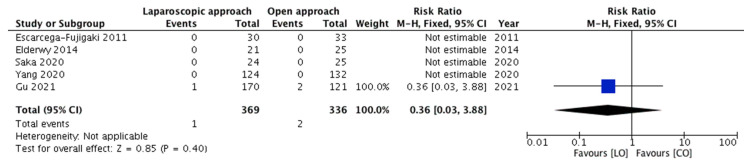
Forest plot comparison between the two treatment groups for the occurrence of testicular atrophy.

All five studies reported this outcome. Testicular atrophy was observed in three children only, and all of them were from the same study. The pooled risk ratio (Figure 4) for the occurrence of testicular atrophy in children belonging to group A compared to group B was 0.36 (95% CI 0.03–3.88), demonstrating no significant difference (*p* = 0.40). As all the events occurred in one study only, assessment of heterogeneity was not applicable.

**Figure 5:**
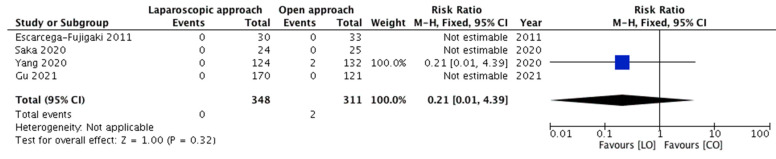
Forest plot comparison between the two treatment groups for the need for redo-orchiopexy after excluding the children with peeping testes.

**Figure 6:**
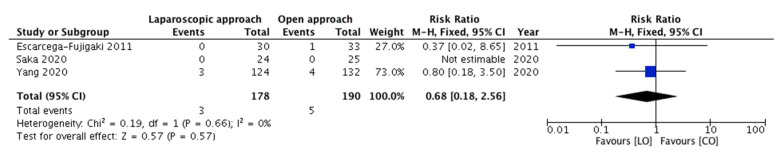
Forest plot comparison between the two treatment groups for the incidence of early postoperative complications after excluding the children with peeping testes.

**Figure 7:**
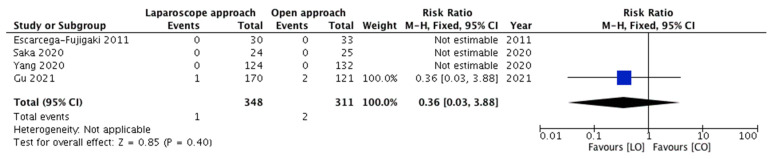
Forest plot comparison between the two treatment groups in regard to the occurrence of testicular atrophy after excluding the children with peeping testes.

Upon excluding the children with peeping testes, the meta-analyses of the outcomes showed no substantial variations in the overall effect and the statistical significance. The pooled risk ratio for recurrence of cryptorchidism (Figure 5), incidence of early complications (Figure 6) and incidence of testicular atrophy (Figure 7) were 0.21 (95% CI 0.01–4.39, 0.32), 0.68 (95% CI 0.18–2.56, 0.57) and 0.14 (95% CI 0.01–2.95, 0.21), respectively.

## 4. Discussion

Cryptorchidism is the most common birth anomaly involving the genitourinary tract in males. At birth, around 3% of full-term and 30% of premature male infants may be diagnosed with cryptorchidism [2]. From the total number of UDT, about 80% descend within 90 days of life [2,4]. After that period, the possibility of spontaneous testicular descent is extremely low. Recent recommendations suggest that optimal timing for orchiopexy, to avoid infertility and testicular cancer is between 6 and 18 months of age [1,2,3,4,5,26]. Although there are many controversies regarding hormonal therapy in boys with non-palpable cryptorchidism, a recent systematic review and meta-analysis showed that hormonal therapy can be effective in some cases and increase the success rate of complete testicular descent [27].

In the majority of the cases of palpable cryptorchidism, CO through the inguinal incision is the standard of surgical care [11]. LO is mainly considered for non-palpable testicles, usually located in the abdominal cavity [11,12]. Several techniques of LO, staged or single-stage, have been described by surgeons across the globe for non-palpable UDT [28,29,30]. The benefits of LO include better visualization, exploration of the whole abdominal cavity, magnification of all structures, an inspection of the contralateral side and the most important dissection above the deep ring/near the kidney is under vision and not blind [19]. Additionally, compared to CO, a wider range of testicular dissection and complete releasing of spermatic vessels can be done, which allows positioning the testes in the scrotum without tension. The possible disadvantages of LO are the learning curve, possible interference with abdominal organs or later formation of adhesions and the need for tracheal intubation [17,18,19]. A recent study showed that tracheal intubation in children older than six months of age, for lower abdominal procedures lasting less than 60 min, should be successfully replaced with laryngeal mask airway [31,32].

Since the first description of LO for palpable cryptorchidism, several studies have reported comparable outcomes with LO as compared to CO [14,17,18,19,20]. In fact, a recent study concluded that the final result is even better when using LO due to the better final position of the testes in the scrotum [21]. The present meta-analyses also depicted the LO approach to be non-inferior to CO in terms of the early and late postoperative outcomes. First, the need for redo-orchiopexy due to the ascent of testes was comparable in both groups. Second, the incidence of early postoperative complications was similar among the children of both the treatment groups. Finally, the rates of testicular atrophy also showed no statistically significant difference. Therefore, LO can be a safe and feasible alternative in palpable UDT as it is associated with all the benefits of laparoscopy.

Moreover, the laparoscopic approach can be extremely useful in high-inguinal and peeping testes [13,18,33]. The advantages of LO may come to the fore in this group of patients as these testes are inconsistently palpable. On the other hand, in these patients, the open approach requires a larger inguinal incision for adequate dissection of the inguinal canal due to the high position of the testes. Moreover, most of these open dissections are blind and may lead to damage of highly sensitive testicular blood vessels or other testicular structures [19,20]. Elderwy et al. in their study compared LO and CO in 46 children with peeping testes. As compared to two patients in the CO group, none of the patients required redo-orchiopexy in the LO group. Therefore, the study concluded that both the techniques of orchiopexy were fairly comparable for the treatment of peeping testes. The findings of less postoperative pain and low redo-orchiopexy rates within the LO group suggested that LO may be a better choice for this subset of the patients [18].

It must also be noted that the success of LO for palpable cryptorchidism depends upon the possibility to pull the testes back into the abdomen through an open internal ring. Gu et al. in their study clearly demonstrated that LO is suitable for children up to two years of age due to the patent deep internal ring. In the case of a closed deep internal ring, it is more complicated to achieve a successful LO because it is hard to pull back the testes into the abdominal cavity [17]. Reopening of deep internal rings may be related to serious damage to the spermatic blood vessels and vas deferens, and subsequently will affect the circulation of testes leading to testicular atrophy. Therefore, the surgeon should select the cases of palpable cryptorchidism that are suitable for LO to avoid complications and to have optimal benefits of the procedure. Besides this, LO is also associated with higher costs compared to CO [18,19,21]. Therefore, it cannot be practiced in resource-challenged settings.

Recently, during the publication of this meta-analysis, we found a similar study by Mentessidou A. et al. [34]. The authors have performed a systematic review and meta-analysis on the same topic and concluded that LO does not have a clear advantage over CO for palpable undescended testes with respect to success and complications, while it is associated with higher cost in all available studies. High retroperitoneal dissection and Prentiss maneuver proposed as the main benefits of LO were found to be unnecessary as well as a likely cause for extra morbidity in a significant number of palpable testes [34]. However, their review has failed to include two relevant studies [17,20] incorporating a total of 340 children (and their events). This can probably be attributed to a sub-optimal literature search by the authors. At least four databases need to be explored for an efficient literature search in systematic reviews [35].

There are a few limitations to this meta-analysis. First, all the studies had a moderate risk of bias. Second, there was a non-uniform reporting of one of the main outcomes (early postoperative complications) in the included studies. Moreover, both the secondary outcomes were selectively reported. In addition, a subgroup analysis based on the age of children was not feasible as only one of the studies sub-categorized the children on the basis of their age. Third, the meta-analysis involves a heterogeneous group of children in terms of the initial position of the testes (canalicular, high-inguinal, and peeping). To overcome this heterogeneity, we performed exclusion analyses for each outcome variable after excluding the children with the extreme form of palpable UDT (peeping testes). Finally, the children included in the present review were operated upon by different surgeons. Various technical modifications have been adopted by surgeons from different parts of the world. Outcome differences can also arise due to a lack of standardized operative techniques among these surgeons.

Despite the above limitations, the present systematic review and meta-analysis is the first to compare the outcomes of children operated via LO versus CO for palpable cryptorchidism. As per the available comparative studies, LO is non-inferior to CO for this subset of children. However, the moderate risk of bias of the included studies limits us to derive an appropriate estimate of the overall effect. The strengths of these studies lie in reporting and external validity while the weaknesses include internal validity and power.

## 5. Conclusions

LO is a safe and feasible alternative to the CO for palpable cryptorchidism. The present meta-analysis revealed similar rates of redo-orchiopexy and the incidence of postoperative complications (both early and late) among both the treatment groups. However, due to a moderate risk of bias of the published comparative studies, well-designed randomized controlled trials with standardized operative approaches need to be conducted for drawing further conclusions.

## Figures and Tables

**Table 1:** Characteristics of the included studies.

Author, Year	Study Design	Sample Size		Mean (SD)/Median (IQR) Age; in Years		Unilateral Cases (%)		Follow-Up Period
		LO	CO	LO	CO	LO	CO	
Escarcega-Fujigaki et al., 2011 [19]	Pro	30	33	Median = 2.3 *		Overall 81% (51/63) were unilateral *		All had follow-up of >6 months ^§^
Elderwy et al., 2014 [18]	RCT	21	25	2 (1.5, 4)	2.5 (1.5, 4)	All unilateral ^†^		1–5.5 years (range)
Saka et al., 2020 [20]	Retro	24	25	2.5 (0.8–10.3) ^#^	2.7 (0.8–11.7) ^#^	63%	80%	Median follow-up = 1.5 and 1.8 years in both groups
Yang et al., 2020 [21]	Pro	124	132	2.3 (0.7–11) ^#^	2.5 (0.8–10) ^#^	87%	83%	Mean follow-up of 1 year
Gu et al., 2021 [17]	Retro	170	121	16.6 (8.4)	18.4 (15.4)	All unilateral		0.5–1 years (range)

* Group-wise characteristics not mentioned. Majority of the children were 1–4 years old; ^#^ Range; ^†^ All cases were peeping testes; ^§^ Median follow-up = 18 months; Abbreviations: Pro—Prospective; Retro—Retrospective; RCT—Randomized clinical trial; LO—Laparoscopic orchiopexy; CO—Conventional open orchiopexy.

**Table 2:** Comparison of the operative duration and cost of the surgery among the groups.

Author, Year	Mean (SD)/Median (IQR) Operative Duration; Minutes		Cost of Surgery
	LO	CO	
Escarcega-Fujigaki et al., 2011 [19]	45 ^#^	38 ^#^	15% higher cost in LO
Elderwy et al., 2014 [18]	40 (40, 45)	40 (35, 45)	25% higher cost in LO
Saka et al., 2020 [20]	U/L = 104 (51−143) B/L = 105 (78−130)	U/L = 95 (62−133) B/L = 114 (89−136)	-
Yang et al., 2020 [21]	62.5 (15.2)	59.0 (13.3)	19% higher cost in LO
Gu et al., 2021 [17]	Age-wise operative durations were compared among the two groups *		-

^#^ Median duration; Range not mentioned; * Patients were divided into four age-groups: 6 months–1 year (I), 1–2 years (II), 2–3 years (III) and >3 years (IV). Operative time was significantly less for the laparoscopic approach among the children belonging to age-groups I and II. On the other hand, the procedural duration for the laparoscopic approach was significantly more for age-groups III and IV. Abbreviations: U/L—Unilateral; B/L—Bilateral; LO—Laparoscopic orchiopexy; CO—Conventional open orchiopexy.

**Table 3:** Downs and Black scale scores for the included studies and the total scores and inter-observer agreement (kappa statistics).

Methodological Assessment by Author 1						
Study	Reporting	External Validity	Internal Validity-Bias	Internal Validity-Confounding	Power	Total Scores
Escarcega-Fujigaki et al., 2011 [19]	7	3	4	3	0	17
Elderwy et al., 2014 [18]	9	1	5	5	0	20
Saka et al., 2020 [20]	9	3	5	3	0	20
Yang et al., 2020 [21]	9	3	5	3	0	20
Gu et al., 2021 [17]	9	3	5	3	0	20
**Methodological Assessment by Author 2**						
**Study**	**Reporting**	**External Validity**	**Internal Valid** **ity-Bias**	**Internal** **Validity-Confounding**	**Power**	**Total Scores**
Escarcega-Fujigaki et al., 2011 [19]	7	3	6	3	0	19
Elderwy et al., 2014 [18]	9	0	5	5	0	19
Saka et al., 2020 [20]	9	3	5	3	0	20
Yang et al., 2020 [21]	9	3	5	3	0	20
Gu et al., 2021 [17]	9	3	5	4	0	21
**Total Scores and Inter-Observer Agreement**						
**Study**	**Rater 1**	**Rater 2**	**Mean**	**Kappa Value**	***p*-Value**	
Escarcega-Fujigaki et al., 2011 [19]	17	19	18	0.926	<0.0001	
Elderwy et al., 2014 [18]	20	19	19.5			
Saka et al., 2020 [20]	20	20	20			
Yang et al., 2020 [21]	20	20	20			
Gu et al., 2021 [17]	20	21	20.5			

## Data Availability

The data presented in this study is available upon request of the respective author.

## Appendix A

PUBMED—((palpable cryptorchidism) OR (palpable undescended testes)) AND ((laparoscopic orchiopexy) OR (laparoscopic orchidopexy));

WOS—((palpable cryptorchidism) OR (palpable undescended testes)) AND ((laparoscopic orchiopexy) OR (laparoscopic orchidopexy));

EMBASE—(‘palpable cryptorchidism’: ti, ab, kw OR ‘palpable undescended testes’: ti, ab, kw) AND (‘laparoscopic orchiopexy’: ti, ab, kw OR ‘laparoscopic orchidopexy’: ti, ab, kw);

SCOPUS—TITLE-ABS-KEY (‘palpable cryptorchidism’ OR ‘palpable undescended testes’) AND TITLE-ABS-KEY (‘laparoscopic orchiopexy’ OR ‘laparoscopic orchidopexy’).

Detailed search strategy

**Database**	**Studies**
PubMed	169
EMBASE	13
Web of Science	72
SCOPUS	59
Total	313
Duplications	110
After Duplication Removal	203
